# What’s down there? The structures, materials and environment of deep-seated slow slip and tremor

**DOI:** 10.1098/rsta.2020.0218

**Published:** 2021-02-01

**Authors:** Whitney M. Behr, Roland Bürgmann

**Affiliations:** 1 Geological Institute, Department of Earth Sciences, Swiss Federal Institute of Technology (ETH), Zurich, Switzerland; 2 Department of Earth and Planetary Science and Berkeley Seismological Laboratory, University of California, Berkeley, CA, USA

**Keywords:** episodic tremor and slip, subduction plate interface, subduction megathrust earthquakes, subduction shear zone rheology, melange belts, slow slip and tremor

## Abstract

Deep-seated slow slip and tremor (SST), including slow slip events, episodic tremor and slip, and low-frequency earthquakes, occur downdip of the seismogenic zone of numerous subduction megathrusts and plate boundary strike-slip faults. These events represent a fascinating and perplexing mode of fault failure that has greatly broadened our view of earthquake dynamics. In this contribution, we review constraints on SST deformation processes from both geophysical observations of active subduction zones and geological observations of exhumed field analogues. We first provide an overview of what has been learned about the environment, kinematics and dynamics of SST from geodetic and seismologic data. We then describe the materials, deformation mechanisms, and metamorphic and fluid pressure conditions that characterize exhumed rocks from SST source depths. Both the geophysical and geological records strongly suggest the importance of a fluid-rich and high fluid pressure habitat for the SST source region. Additionally, transient deformation features preserved in the rock record, involving combined frictional-viscous shear in regions of mixed lithology and near-lithostatic fluid pressures, may scale with the tremor component of SST. While several open questions remain, it is clear that improved constraints on the materials, environment, structure, and conditions of the plate interface from geophysical imaging and geologic observations will enhance model representations of the boundary conditions and geometry of the SST deformation process.

This article is part of a discussion meeting issue ‘Understanding earthquakes using the geological record’.

## 1. Introduction

**Figure 1.**
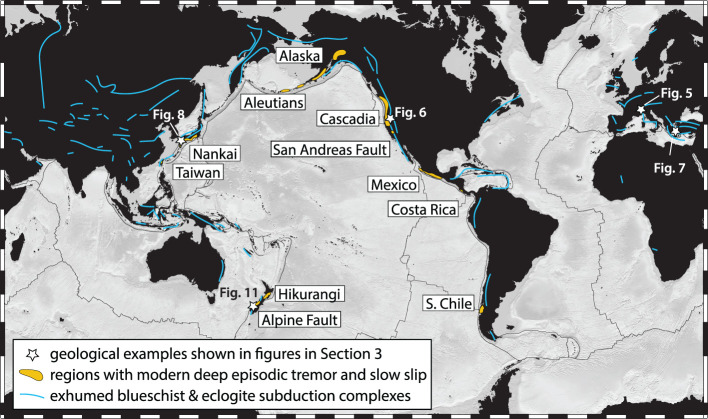
Global distribution of regions with modern deep slow slip and tremor along with exhumed blueschist and eclogite facies subduction complexes. Locations of geologic sites presented in figures in §3 are also labelled. (Online version in colour.)

What was long considered to be bothersome seismic noise and long-period errors in geodetic time series turned out to be one of the exciting discoveries in the Earth Sciences in recent decades: episodic slow slip events (SSEs) and associated tremor signals originating deep on the plate interface of the Nankai, Japan, and Cascadia, Canada and USA, subduction zones (figure 1) [1–3]. Ever since their original discovery, we have been wondering what things look like down there; that is, what are the geologic materials and structures of slow slip and tremor (SST)?

**Figure 2.**
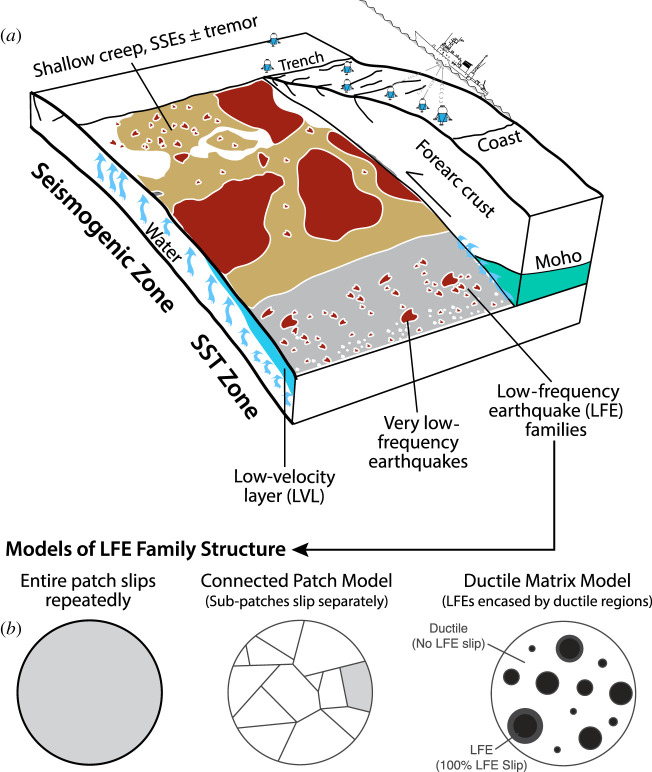
(*a*) Summary schematic of the geophysical view of slow slip and tremor (SST) and informed by geophysical imaging, source seismology and geodesy. Earthquake ruptures in the seismogenic zone, and low frequency earthquakes (LFE) and very low frequency earthquakes in the SST zone release seismic energy (red patches). The plateinterface away from the seismic patches slip by aseismic creep, often in episodic slow slip events. Slow slip in the SST zone (grey) is illuminated by tremors. LFE source patches are clustered in families that are sometimes aligned in the plate convergence direction. Fluid pressure is likely high along much of the plate interface and reaches lithostatic levels in the SST zone under the mantle wedge corner and forearc crust. Modified from [4,5]. (*b*) Bursts of events in LFE families may reflect repeated failure of the same patch, connected sub-patches or more distributed source patches in a ductile matrix (based on [6]). (Online version in colour.)

**Figure 3.**
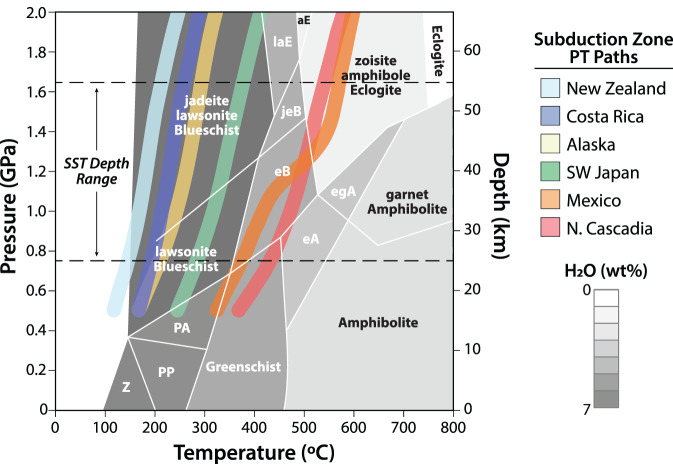
Calculated pressure-temperature profiles for the top of the slab for several SST-hosting modern subduction zones, superimposed on metamorphic facies and weight per cent water release. Subduction zone geothermal gradients come from thermal models of [7], modified according to [8]. Water content estimates are from [8]. Metamorphic facies abbreviations are as follows: Z, zeolite; PP, prehnite-pumpellyite; PA, prehnite-actinolite; eB, epidote blueschist; jeB, jadeite epidote blueschist; laE, lawsonite eclogite; aE, amphibole eclogite; eA, epidote amphibolite; egA, epidote garnet amphibolite. (Online version in colour.)

The ‘SST zone’ is located within depth and temperature ranges of about 25–55 km and approximately 350–550^°^C, which are conditions commonly associated with hot and young subducting oceanic plates, downdip of the ‘seismogenic zone’ hosting regular earthquakes of up to *M* > 9 (figures 2 and 3) [4]. Tremors at similar pressure and temperature conditions are also found in continental plate boundary faults, including the strike-slip San Andreas Fault in California [9], the oblique right-lateral Alpine Fault in New Zealand [10], and the continental collision zone in Taiwan [11] (figure 1). Downdip of this zone, plate boundary deformation appears to be steady, accommodated by ductile shear. Updip of the SST zone, some subduction zones abut locked sections of the seismogenic megathrust (e.g. Kii Peninsula, Japan), some host larger, longer-lasting and less frequent SSEs (e.g. Tokai and south Shikoku in Japan, Guerrero in Mexico), while others feature a wide, steadily creeping zone with little if any tremor, forming an apparent gap between the SST zone and locked asperities (Cascadia). In some subduction zones, tremors and slow slip are also found at intermediate and shallow depths [12,13], suggesting that conditions for SST can exist at all depth ranges of subduction thrusts. Here we focus on the deep-seated SST region of subduction zones and their potential analogues exhumed from greenschist-, blueschist- and eclogite-facies environments.

There is abundant evidence that indicates a close temporal and spatial association of tremor and slow slip [14,15]. Tremors represent enduring low-frequency seismic signals generally interpreted as being a direct byproduct of otherwise slow and aseismic slip. While on average the seismic moment released by tremor amounts to only approximately 0.1% of the associated slow slip [6,16], tremor is generally considered as a direct marker of the spatio-temporal evolution of slow slip during an SSE. However, while tremor and slow slip are closely associated, they are not always exactly coincident in space and time. Slow slip may occur without tremor, and smaller SSEs may be indicated by tremor transients, but lack a resolvable geodetic signal [17,18]. The study of small (*M* < 2.5) low-frequency earthquakes (LFEs) and M 3–4 very low-frequency earthquakes embedded in the tectonic tremor signal allows for more detailed investigation of underlying source properties and the spatio-temporal distribution of fault slip. Thus, accurate locations and source properties of tremors and LFEs are essential ingredients for improved understanding of the slow slip process.

So, what are the rocks and structures preserved in the geologic record that could represent deep tremor and slow slip? An essential aspect of understanding the SST deformation process is the examination of rocks exhumed from SST source depths, which are exposed in subduction complexes in a wide range of tectonic settings around the globe (figure 1). Exhumed subduction complexes can contain slivers of the downgoing slab, the upper plate, and the interface shear zone itself. Through carefully probing these exposures to distinguish subduction versus exhumation features, we can identify ‘snapshots’ of the SST source region captured at a range of depth and temperature conditions, and use them to provide key insights into lithological and rheological contrasts, short- and long-time scale deformation processes, interplay between deformation and metamorphism, and fluid migration patterns. Observations focused on this topic thus far suggest that rocks preserve a record of both long-term strain accumulated over millions of years, as well as punctuated transient deformation set up in regions where lithologies are mixed, fluids are abundant, and fluid pressures are near-lithostatic. Here we first provide an overview of inferences made from geophysical observations about the environment and deformation processes in the SST zone and then we review knowledge gained from geological studies of relevant analogue field examples. Improved models and understanding of deep slow slip processes in plate boundary faults will require drawing on both types of observations.

## 2. Geophysical observations of SST environment and source

Deep-seated SST represents only a small part of the wide spectrum of seismic and aseismic slip processes that we now recognize to occur across the whole width of the subduction plate interface [5,19,20]. In this section, we review what has been learned about the environment, the kinematics and the dynamics of SST from geophysical imaging and geodetic and seismologic observations. We focus on aspects that can potentially be related to findings obtained from geologic analogues of the deep SST source region.

### (a) The environment of deep SST

**Figure 4.**
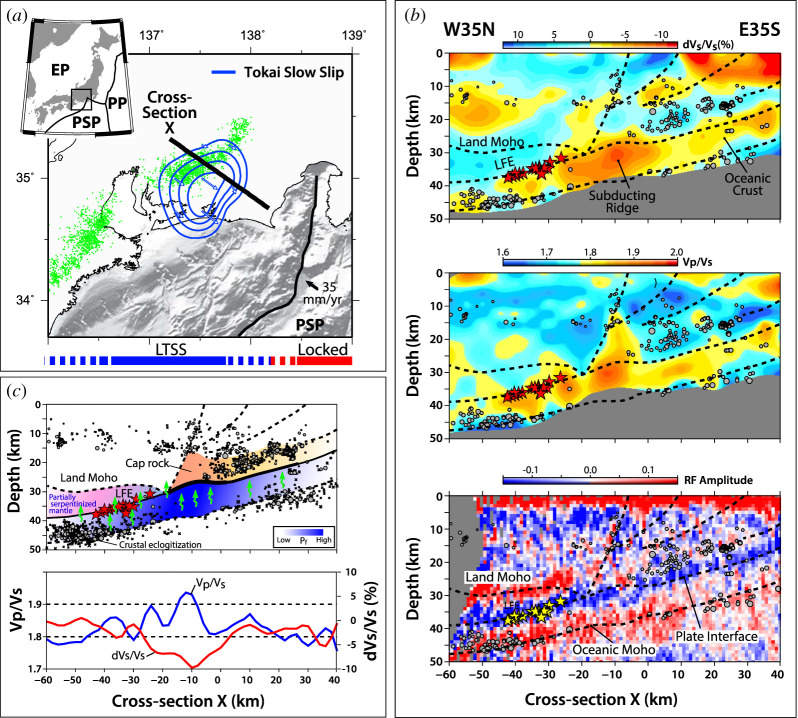
Seismic imaging of Philippine Sea Plate subduction thrust hosting slow slip and tremor beneath Tokai District in central Japan. (*a*) Map showing slip contours of long-term SSE (blue),low frequency earthquakes (green circles), and northwest-southeast profile line (black). (*b*) Top: Depth section of S-wave velocity perturbation (d*Vs*/*Vs*) from seismic tomography. Centre: Ratio of P and S-wave velocities (*Vp/Vs*). Bottom: Receiver function results (RF amplitude) highlighting sharp changes in seismic velocities that illuminate the plate interface and the continental and oceanic Moho. (*c*) Top: Schematic interpretation of seismic structures. Blue shades in subducting oceanic crust reflect fluid pressure variations. Green arrows denote potential fluid pathways in the subduction zone. Bottom: Profile showing variations of d*Vs*/*Vs* and *Vp/Vs* within the subducting oceanic crust. Relocated hypocentres are plotted for events within 10 km of the cross-section with grey circles indicating regular earthquakes and red stars indicating LFEs. The deep ETS zone hosts LFEs and M6 slow slip events recurring every few months [26]. An intermediate-depth section of the plate boundary hosts long-term M7 slow slip events (LTSS) that last for years and shorten the deeper ETS recurrences. The updip locked zone fails in M8 megathrust ruptures every approximately 150 years. (All images provided by A. Kato based on [25].) (Online version in colour.)

In subduction zones, deep SST are found near and updip of the mantle wedge corner, where the subducting oceanic crust interfaces with the overriding forearc crust and, often serpentinized, continental mantle (figure 2). The section of the subduction thrust hosting SST lies near the top or within a zone of low seismic velocities and very high ratios of the *P*-wave and *S*-wave velocities (*Vp/Vs*), the low-velocity layer (LVL) [21–24]. For example, in central Japan, Kato *et al.* [25] find an LVL with low seismic velocities and high *Vp/Vs* ratios, both along the zone of tremor and long-term SSEs without tremor (figure 4). Here, a zone of frequent ETS events appears on the deeper plate interface beneath the partially serpentinized mantle wedge, while more enduring years-long SSEs without tremor occur farther updip, below the forearc crust. In the Cascadia, southwest Japan, Costa Rica, New Zealand and Alaska subduction zones, the maximum *Vp/Vs* regions and SST zones also appear to roughly coincide with the intersection of the subducting plate with the mantle wedge corner [27].

There continues to be some debate about the makeup and state of the LVL. Based on seismic data from northern Cascadia, Hansen *et al.* [28] argue that the 3–4 km thickness of the LVL, its high *Vp/Vs* ratio and its limited downdip extent support the inference of the SST zone consisting primarily of uppermost oceanic crustal rocks at very high fluid pressures. By contrast, Abers *et al.* [29] and Calvert *et al.* [30] favour the LVL being dominated by underplated metasedimentary rocks. The subduction interface may be fluid saturated and frictionally weak across much of its downdip width thanks to fluids released by the downgoing slab; however, Audet *et al.* [31] use onshore–offshore receiver function data to show that the LVL thins offshore and does not extend to the locked section of the megathrust. High electric conductivity is also found in the SST zone of the Cascadia subduction zone, but not in the locked updip section of the megathrust [32]. Delph *et al.* [33] find zones with a much thicker (approx. 10 km) LVL in both the northern and southern Cascadia subduction zone, which appear to correlate with increased tremor rates and seismicity in the underlying slab. This may suggest that thick underplated sediments invaded by slab-derived fluids are related to increased tremor occurrence in those zones [33]. However, from a geophysical perspective alone, the exact makeup of the LVL remains uncertain [31].

Seismic receiver function studies find an anisotropic fabric in the LVL plunging at an oblique angle to the plate interface both in Cascadia and Mexico, suggesting distributed shear and the development of high strain fabrics [24,28]. This supports the idea that the LVL reflects the development of an increasingly thick, fluid-rich and overpressured shear zone hosting both aseismic and ultimately tremor-producing slow slip. LFEs appear to locate within the LVL [31], but the exact vertical distribution of the tremor sources and deformation in the LVL is not well established.

High *Vp/Vs* ratios of 2–3.5, and equivalently Poisson’s ratios of 0.33–0.46 (table 1), are consistent with strongly elevated pore fluid pressures in porous (up to 4% porosity) and highly strained metabasalts and metasediments of the subducting oceanic crust [34,35]. The observed velocity ratios suggest pore fluids under near lithostatic pressure, but *Vp/Vs* is also increased in rocks with high fracture densities [36]. The fluids are made available by prograde metamorphic dehydration reactions in the subducting oceanic crust [37]. At these temperatures and pressures, water is a supercritical fluid and is a factor of approximately 10 less viscous than near-surface water. Sustaining near lithostatic fluid pressures requires a capping seal with very low vertical permeability [23,34]. This seal may be formed in part by the strongly sheared fault-zone rocks of the plate interface whose permeability is likely strongly anisotropic [38]. Nakajima *et al.* [39] and Wells *et al.* [40] relate the distribution of tremor to the degree of metamorphism and distribution of fault zones in the hanging wall of the megathrust, respectively, which appear to provide some control on lower-crustal permeability and thus on the fluid pressure in the LVL.

**Table 1.** Basic definitions of common terms used herein

phenomenon	definition and notes
slow slip and tremor (SST)	General term used here for plate boundary slip transients that are commonly associated with observable surface deformation over days to years and that are illuminated by low intensity seismic emissions known as tectonic tremor
slow slip events (SSEs)	Aseismic fault slip transient with durations ranging from minutes to decades. Also referred to as slow earthquakes, silent earthquakes and creep events.
episodic tremor and slip (ETS)	SSEs with abundant tremor, typically months or less in duration, found at and around the mantle wedge corner of some subduction zones. More narrowly defined type of periodic slow slip transients with tremor based on the characteristics found in southwest Japan and Cascadia-type localities.
low frequency earthquakes (LFE)	Small and short low frequency (2–8 Hz) seismic events contained in tectonic tremor signal indicative of fault slip. See table 2 for details.
very low-frequency Earthquakes	Similar to LFEs but lower frequency (less than 0.1 Hz), longer duration (10–200 s) and larger (M 3–4).
subduction interface	The region between the subducting slab and the overriding plate in subduction zones that accommodates differential motion between the two plates. It can be a fault, an anastomosing fault zone or a finite-width shear zone.
tectonic underplating	The progressive transfer of material from the top of the subducting slab to the upper plate, and the associated down-stepping of the subduction interface into deeper levels of the downgoing plate. Underplated material may derive originally from the downgoing slab, but can also be eroded updip from original or previously accreted material in the overriding plate.
asperity	Defined here as a feature on a fault that exhibits different rheological properties from surrounding regions. Often used to describe areas that will eventually fail by seismic rupture.
melange belt	A general term used here to refer to a fault- or shear- zone with rock types that exhibit strong competency contrasts such that a ‘block-in-matrix’ texture is visible at the outcrop or larger scale. Does not necessarily require the presence of exotic blocks or large-scale mechanical mixing.
pressure solution creep	A deformation mechanism common in rocks in subduction (and other fluid-rich) environments. It involves dissolution of minerals along grain boundaries in areas of relatively high differential stress, accompanied by mass transfer within the fluid phase and eventual deposition of minerals in regions of relatively low differential stress. Produces strong foliations at high strains, known as pressure solution cleavages or cleavage microlithon fabrics. Also referred to as dissolution-precipitation creep or volatile-assisted diffusion creep.

Audet & Bürgmann [41] find that *Vp/Vs* ratios in the lower crust above the tremor zone are substantially reduced from typical forearc values, consistent with the addition of quartz precipitates (5–15% by volume) to the upper plate [42,43]. This is consistent with the idea that fluids are channelled updip in the permeable plate interface below the serpentinized forearc mantle and are released upward at the mantle wedge corner into the more permeable crust [42]. In addition, there appears to be a correlation between ETS recurrence intervals and upper-plate silica enrichment, suggesting that increasing quartz-vein mineralization from slab-derived fluids reflects more rapid development of fluid overpressure and therefore shorter recurrence times. This correlation appears to hold both for a number of different subduction zones and for the observed systematic decrease in the recurrence time of ETS with increasing depth of the plate interface in the northern Cascadia subduction zone [41]. These results suggest cycles of slow slip episodes, dilatancy and healing that produce rapid changes in permeability and fluid pressure.

To achieve high fluid pressure, pathways for fluid transport along the megathrust or into the overlying mantle wedge or forearc crust may be intermittent [41]. There is some observational evidence, from temporal changes in gravity, seismic velocity, seismic attenuation, seismicity and state of stress, that indicates pressure changes and fluid transport along and across the fault zone associated with slow slip episodes. In the Tokai District in central Japan (figure 4), Tanaka *et al.* [44,45] find absolute gravity changes during two approximately 5-year-long slow-slip episodes, invoking cycles of fluid pressure and fault zone permeabilities of 10^−18^–10^−15^ m^2^. In the northern Cascadia subduction zone, Gosselin *et al.* [46] document seismic velocity changes that may reflect seal breaching and fluid flow in permeable pathways within and away from the megathrust, resulting in transient fluid pressure drops of 1–10 MPa. Nakajima *et al.* [47] explore temporal changes in slip rate, seismicity and seismic attenuation along the approximately 50 km deep megathrust of the Philippine Sea in central Japan to infer cyclic drainage episodes from the megathrust. Attenuation and seismicity in the overriding plate are enhanced within a few months following an SSE, suggesting permeable pathways into the upper plate from near the updip edge of the slow slip zone [47]. In the relatively shallow SST zone of the Hikurangi subduction zone in New Zealand, Warren-Smith *et al.* [48] document time-dependent variations in the state of stress in the underlying, overpressured oceanic crust from focal mechanism data. The data are interpreted as being the result of inter-SSE rise and co-SSE drop of fluid pressure in the overpressured zone by several MPa, reflecting multiple cycles governed by fracture and healing processes in the plate boundary zone [48].

### (b) SST source characteristics

**Figure 5.**
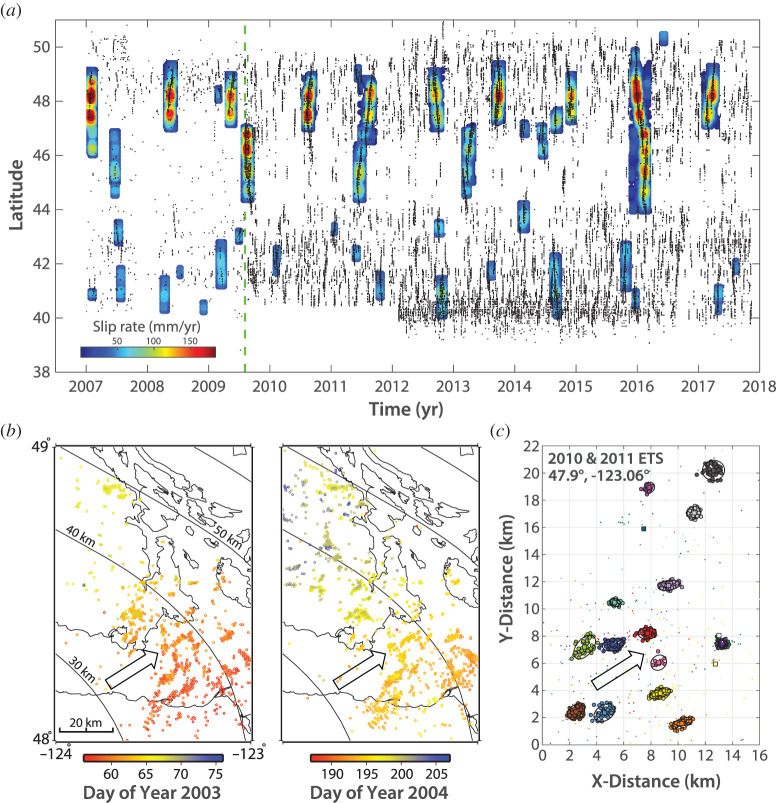
Geodesy and source seismology illuminate the hierarchy of ETS and LFE on Cascadia subduction zone. (*a*) Spatio-temporal distribution of slow slip events (colour contours indicating slip rate) and tremors (black dots) along the ETS zone of Cascadia (based on [49]). (*b*) Spatio-temporal distribution of tremor sources during two ETS events in northern Cascadia. Tremor clusters light up many of the same source patches in recurring events (based on [50]). (*c*) LFEs clustered in LFE families that failed during two ETS in 2010 and 2011 (also shown in (*a*)) (based on [6]). Open arrows in (*b*) and (*c*) indicate the plate convergence direction. (Online version in colour.)

Inversions of geodetic time series (from GPS, tilt- and strainmeters) and the spatio-temporal distribution of tremors paint a picture of highly dynamic slow slip processes on the deep plate interface below the seismogenic zone (figure 5). These slow slip transients span a wide range of many orders of magnitude in dimension, rate and duration (see table 2 for typical values and related references). Here we summarize the slow slip behaviour in the SST zone including the macroscale description of large and small SSEs, the mesoscale characterization of the transient slip dynamics on the rupture surface during an SST episode, and finally the nature of rapidly repeating failures of individual LFE clusters. The spectrum of geophysically visible SST behaviour ends at the scale of individual LFE failures with 100 s of m dimension, which is larger than a typical outcrop examined by geologists. Nonetheless, interesting connections can be made between the properties of observed SST failures and the geologic analogues.

**Table 2.** Summary list of relevant deep SST-zone properties from geophysical and laboratory constraints.

parameter	constraint or observable	typical values and notes	references
depth	tremor locations and SSE modelling	25–50 km	[27]
		(to >60 km in a few areas) [51,52]	
lithostatic overburden	from depths (assuming average density 2700 kg m^−3^)	650–1200 MPa	
temperature	from thermomechanical modelling	$350\text{–}550^{\circ }\text{C}$	[34,53]
major hydrous mineral breakdown reactions	thermal and petrological modelling	ultramafic rocks: antigorite, chlorite	[8,54,55]
		mafic rocks: lawsonite, chlorite, glaucophane	
		metasediments: phengite, lawsonite, chlorite	
geometry of low-velocity layer (LVL) hosting ETS	seismic tomography and receiver function analysis	∼4 ± 1 km-thick	[25,27,31,33]
		(between higher-velocity layers, thinning updip and disappearing at approximately 50 km depth. Can be ≫ 5 km in places)	
fluid pressure, effective normal stress	from *Vp*/*Vs* = 2–3.5 found in receiver function studies (lower-resolution tomography finds somewhat lower values)	∼ lithostatic	[22,27]
		(effective normal stress ∼1 MPa or less)	
porosity	from *Vp/Vs*, electrical conductivity	3.3–4%	[34]
Poisson’s ratio	from *Vp/Vs* $\nu =\frac{1}{2}\frac{\left(Vp^{2}-2Vs^{2}\right)}{\left(Vp^{2}-Vs^{2}\right)}$	0.33–0.46	
fluid viscosity	laboratory	∼10^−4^ Pa s	[34]
permeability	*Vp/Vs* contrast across plate interface and laboratory measurements	<10^−21^ m^2^	[23,38,56,57]
		(permeability is likely highly anisotropic and time dependent through the ETS cycle)	

#### (i) Macroscopic: large-scale SSE characteristics

Inversions of geodetic measurements allow for characterizing the macroscopic dimensions, slip and other kinematic source properties of large-scale SST events. One outstanding feature of these SSEs is the minute amount of slip (few centimetres) that even the largest (more than 100 km along arc extent) events accommodate (table 2). Despite the small amounts of slip, SST events are frequent enough in some places (e.g. Cascadia) that they are estimated to take up between 60 and 100% of the total slip budget [58]. Similar-sized ETS events frequently recur every few months to a couple of years, and thus many dozens of events have now been observed in the well-monitored subduction zones in Cascadia and Japan. The small slip per event also means that the ETS events have very low stress drops of less than 100 kPa, compared to the 1–100 MPa range of regular earthquakes. SSEs in the tremor-producing zone appear to be limited to a seismic moment equivalent to Mw 6.7, slowly released over the course of a few-weeks-long episode. In contrast to the classic ETS zones hosting weeks-long slip and tremor events (Cascadia, southwest Japan), some deep SST source regions (e.g. New Zealand, Bungo Channel and Tokai in Japan, Mexico and Alaska) exhibit many months- to years-long episodes of accelerated slip amounting to Mw 7 events. In some cases, these long-lived SSEs occur updip of shorter duration ETS source regions (e.g. [25,59–61]), suggesting that relatively modest changes in fluid pressure, thermal conditions or shear zone composition can lead to such variable fault behaviour. Careful analysis of both geodetic and tremor datasets suggests that in some of these cases (Mexico, Alaska, southwest Japan), the slip during long-term SSEs and at other times is composed of many short-duration events illuminated by the tremor activity (e.g. [62,63]). This suggests that the slow-slip process in general may represent the accumulation of many smaller slip events.

In addition to their small slip and low stress drop, SST stand out by the slow propagation (approx. 10 km d^−1^) of their primary slip front and thus long duration of their rupture (figure 5*a*). The apparent ease of growth and frequent repeat of such large-scale failures suggests a very effective means of stress communication and slow rupture propagation. Like regular earthquakes, SST have a nucleation zone from which they often propagate updip and then either bilaterally or unilaterally along strike of the subduction zone [62,64–66]. While the along-arc propagation of the main slip front is slow, slip-parallel migrations of tremors propagate both updip and downdip hundreds of times faster (30–200 km h^−1^). This might involve interaction of the slowly laterally migrating slip front with slip-parallel linear structures on the fault or rapidly propagating fluid pressure pulses along structural features elongated in the dip direction [66–68]. In some cases, the nucleation and growth of SST can be rather complex. For example, Bletery & Nocquet [69] consider a 2013 SST in which tremor and GPS data suggest initial nucleation in three different spots followed by growth and coalescence, over the course of three weeks. As the three slip fronts approach each other and merge, the rate of moment release substantially increases, suggesting that coalescence of multiple SST can lead to a more energetic event [69]. Frank *et al.* [70] evaluate the slip evolution of a long-term SSE inferred from GPS time series and LFE recurrence intervals in the Guerrero subduction zone to develop a conceptual model of updip migrating pore-pressure pulses modulating the slip and strength of the fault zone. As the SSE develops updip of the LFE source region, it loads and accelerates downdip LFE activity and slow slip. Fracture reactivation and increased anisotropic permeability in the LFE zone leads to a transient decrease in pore pressure that decelerates the LFE activity as the pore-pressure pulse migrates updip, even as the long-term SSE continues in the updip section. This case study supports the idea that fluid pressure cycles and fault valve (e.g. [71,72]) behaviour control the spatio-temporal distribution of slip in the SST zone.

As pressure, temperature, permeability and other conditions and properties vary both in the downdip and along-arc direction, there is interest in resolving systematic effects of such parameters on the SST behaviour [41]. Wech & Creager [65] use the tremor activity in Cascadia to find a systematic decrease in size and increase in frequency of events with increasing depth. A similar first-order transition to more episodic slip behaviour is found in the Nankai subduction zone and in Mexico [73,74]. Wech & Creager [65] put forward a model invoking a cascading stress transfer process, Audet & Bürgmann [41] suggest that the systematic decrease of recurrence interval with depth is governed by temperature dependent silica precipitation and healing processes, which reduce fault zone permeability and thus accelerate overpressure development and shorten the time to the next SST failure. Idehara [75] carry out a systematic analysis of changes in the temporal clustering of tremors to evaluate such spatio-temporal patterns in tremor sequence duration and recurrence intervals in a handful of global tremorgenic subduction zones, finding both downdip and lateral variations. More such comprehensive explorations of variable ETS behaviour in the context of varying SST zone conditions should improve our understanding of the underlying mechanics and hydrology of the SST phenomenon.

#### (ii) Mesoscale: spatio-temporal tremor distribution and SST slip variability

The distribution of tremors in space and time provides detailed information about the structure and dynamics of ETS. It appears that tremor sources persistently illuminate many of the same patches in the recurring ETS events, suggesting inherent structural or lithological differences in the tremor-producing portions of the SSE rupture. Ide [76] highlights apparent alignments or striations in the tremor source distribution in Japan, which appear to align with the direction of plate motion but also past plate convergence directions. This suggests that the striations reflect plate interface structures that could have developed from subducting plate structures, such as seamounts. Similarly, Armbruster *et al*. [50] show that the same clusters of LFEs light up in repeated SST, suggesting that these are persistent features of the plate interface (figure 5*b*).

Once the main slip front of an SST rupture has passed, dynamic bursts of tremor activity [64,77] and slow slip [78] continue in its wake, for many days. During this period, coherent migrations of LFEs and tremor on slipping portions of the fault are observed, suggesting secondary slip transients with a range of dimensions, propagation speeds and directions, moments, stress drops and other characteristics [66,68,77–80]. The secondary slip fronts start within about 1 km of the main tremor front, and propagate at variable rates backwards, forward and parallel to the main front [66,80]. Bletery *et al.* [80] used cluster analysis to catalogue more than 1000 of these secondary tremor and LFE migrations contained in Cascadia ETS, lasting up to approximately 30 h, to systematically inventory their source properties. They find that short-duration secondary slip fronts dominantly propagate along dip while the more enduring ones mostly propagate along strike. Peng *et al.* [77] find many smaller-scale subevents propagate at 10–60 km h^−1^ right behind and parallel to the main front of tremor migration, which may or may not be aligned with the dip direction. They thus conclude that even though the SST zone may have a slip-parallel anisotropic fabric it does not control the orientation of the main front or strongly influence the migration pattern of the secondary fronts. The larger-scale and somewhat slower tremor migrations occur further behind the main front and appear to continue across portions of the fault without many tremor sources [64,77]. These later migrations advance at 10–20 km h^−1^ [64,68], 25–50 times faster than the main SSE front, and their slip is faster by a similar ratio. As opposed to the initial set of secondary tremor bursts, events in these larger-scale secondary tremor migrations are strongly tidally modulated [81]. The tidal sensitivity increases over the course of several days, suggesting that the fault weakens as SST slip grows [82]. Thus, it appears that some aspect of the SST faulting process limits the main slip front growth and speed but permits the secondary fronts to propagate and slip faster and to weaken and become increasingly sensitive to tidal stress.

#### (iii) LFE source characteristics

LFEs are small seismic events contained within tremor, first recognized by Katsumata & Kamaya [83] in southwest Japan. They represent barely observable seismic signals extracted from continuous waveform data, which are characterized by lower frequencies (more than 1 Hz) and longer durations (0.2–0.5 s) than those observed for ordinary microearthquakes [84,85]. It is possible that localized, near-source attenuation of seismic waves, which may be the result of high pore-fluid pressures and rock damage in the LVL, could cause the bandlimited nature of LFEs through the depletion of high frequencies [86,87]. LFEs with a maximum size of about Mw 2.5 have approximately constant durations and appear to break asperities of similar dimension (1 km), suggesting that moment variation is dominated by differences in slip [88]. LFE focal mechanisms and polarizations are consistent with a double-couple source, originating from areas in the plate interface that are stationary in space and persist for decades [50,89,90].

An important characteristic of LFEs is their repetitive nature; each LFE source in space (LFE family) can generate hundreds of events during each SST, interpreted as either the repeat failure of a single asperity or adjoining failures within a relatively tight cluster of asperities (figure 5*c*) [6,9,18,91]. Recurrence intervals in a family during an SST can be as low as a few seconds. Such rapid repeat failures challenge standard models of failure cycles involving healing and restrengthening of a slip surface. Chestler & Creager [6] argue that each LFE family typically hosts multiple separate patches (figure 2*b*). Thus, while estimates of slip per LFE are of the order of 0.1 mm, assuming single-patch failures [6], slip could reach a mm if there are approximately 10 patches in a family and more if those patches are spatially separated in a ductile matrix [6] (figure 2*b*). Stress drops are thought to be approximately 10 kPa [88,92], but Chestler & Creager [6] suggest that stress drops could be up to 1 MPa, getting close to values for regular earthquakes, if LFEs in a family have smaller slip areas and are more spaced out on the family patch (figure 5*c*). Given that geological observations indicate that subduction thrusts have finite thickness increasing with depth (see below), the LFE families could also be distributed in a three-dimensional volume of ductile material, thus allowing for less slip and lower stress-drop per LFE [6]. LFEs and tremor only release a tiny fraction (approx. 0.1%) of the otherwise aseismic plate-interface slip in the ETS zone [6]. This suggests that LFE family clusters represent small areas in the fault zone that are mechanically distinct from their aseismically shearing surroundings, representing local anomalies in lithology, metamorphic assemblage, fluid pressure and/or permeability.

In addition to LFEs, distinct very low-frequency earthquakes have been detected in broadband seismic records at even lower frequencies of 0.02–0.05 Hz [93]. Occurring within tremor zones, both deep and near-trench, they seem similar to LFEs and are also the result of shear slip on the plate interface, but they have longer durations (10–200 s) and larger magnitudes (M 3–4) [14,94]. While many of these more enduring slow earthquakes appear to be contained in tremor sequences, they can occur independently, separated from tremor in both space and time [95]. Even though these very low frequency sources appear to be clearly distinguished from LFEs, Kaneko *et al*. [96] suggest that this separation is potentially an artefact of Earth’s microseism noise hiding signals in the intervening frequency range (0.05–1 Hz). Thus the different seismic and aseismic slip phenomena observed in the SST zone may be parts of a common broadband slow slip process [20,97]. Improved seismological and geodetic observations with higher spatio-temporal resolution and over a broader frequency range would help resolve further details of the complex slip behaviour in the SST zone.

### (c) Scaling and probing the mechanical properties of SST

To better understand the underlying mechanics of slow slip and regular earthquakes, there has been much interest in the scaling relationships of SSEs, in particular that between their moment and duration [49,98,99]. Ide *et al.* [98] suggest that the moment of SSEs scales linearly with their duration over a wide range of scales from individual LFEs to the largest SST events. This is in contrast with the systematic duration-cubed scaling of regular earthquakes. However, several recent studies [49,100–102] find that such a systematic difference does not hold when considering SSEs from a particular environment or of a different type. Thus, ETS, secondary slip migrations, very low-frequency earthquakes, LFEs and regular earthquakes may all feature similar, pulse-like rupture propagation and their rupture velocities and stress drops vary with the size of the event. Nonetheless, there is a large and real gap in detection of fault slip processes between the two proposed scaling relationships for SSEs and regular earthquakes, suggesting that earthquakes and slow slip phenomena are two distinct fault slip processes that seem to indicate a different geological context [79]. More quantitative studies of scaling relations of SSE across their full spectrum are needed to improve our understanding of similar and dissimilar dynamics of slow and fast ruptures under different conditions.

Evidence supporting the idea that fluid pressures are very high, stress levels are low, and faults are frictionally weak in the SST zone comes from observations of triggering of slow slip and tremor by seismic waves and Earth’s tides [19,81,94,103–107]. Tremors are quite easily triggered and their amplitude modulated by few-kPa shear stress cycles from passing surface waves of remote earthquakes [19,103,104]. Similarly, small tidal shear-stress changes produce substantial modulation, while the normal-stress cycles produce a more modest response, interestingly indicating raised rates during times of increased fault-normal compression [107]. The tidal shear-stress response appears consistent with rate-dependent friction at extremely low effective normal stress, whereas purely aseismic shearing of various mineralogies and power-law or exponential viscous deformation mechanisms does not appear to allow for driving such a response [108]. Considering an undrained fault model, Beeler *et al.* [109] suggest that the observed tremor response reflects low intrinsic friction, low dilatancy and lithostatic pore fluid pressures. Houston [82] finds that the modulation of tremors becomes stronger as slow slip accumulates during an SSE. This indicates that the plate interface has an intrinsically low dynamic friction coefficient (less than 0.1), is at near-lithostatic fluid pressure, and further weakens during continuing slow slip associated with secondary slow slip fronts [82]. Examination of tidal modulation of individual LFE families shows strong spatial variability in the correlation with tidal stress in addition to systematic temporal changes in which tidal correlation increases with time during secondary slip fronts and transitions from correlation with stressing rate to correlation with stress amplitude [107]. These studies show that detailed examination of the response of tremors and slow slip to very modest external stressing cycles allows for characterizing laterally heterogeneous and time-dependent physical fault-zone properties.

## 3. Geologic observations of SST environment and source

Subduction complexes exhumed from depths similar to the SST source region (approx. 25–50 km) occur on several continents and in a wide range of tectonic settings (figure 1) [110–112]. Around the circum-Pacific, rocks from this source depth are dominantly oceanic in affinity and crop out in the inboard parts of long-lived accretionary prisms [113–117]. Within the Mediterranean orogens, several exhumed subduction complexes occupy the footwalls of large-scale metamorphic core complexes, and consist of intercalated oceanic and continental-affinity rocks representing subduction of rifted continental fragments and intervening small ocean basins [118–120]. Several oceanic-affinity subduction complexes also crop out in the internal zones of continental collision zones (e.g. the Alpine–Himalayan mountain belts), recording early stages of oceanic subduction and accretion prior to continental collision [121–123]. The mechanisms of exhumation of these subduction complexes are debated, but likely involved some combination of buoyancy- or pressure-driven return flow along the top of the subducting slab, and upper plate extension driven by surface elevation gradients or slab rollback [115,124–127].

Relating features preserved in these subduction complexes to processes in the modern SST source region has several challenges. These include firstly that subduction complexes exhumed from this depth range are too deep to be exhumed simply by erosion, and instead always involve some tectonically driven exhumation process. In many cases, the exhumation path is along a warmer geothermal gradient than the prograde path, so subduction-related structures and metamorphic relationships can easily be obscured. Secondly, even where exhumational overprinting is weak, individual packages of rock within these complexes record subduction and tectonic accretion over several million to tens of millions of years, thus aggregating deformation over much longer timescales than individual SSEs. Thirdly, there are debates as to whether the rocks we see exhumed to the surface are representative of ‘average’ subduction zones. van Keken *et al.* [128], for example, suggested that exhumed blueschist-eclogite terranes may reflect only young oceanic lithospheric slices based on a data compilation by Penniston-Dorland *et al.* [110] indicating that rocks were hotter than thermomechanical model predictions. A more recent compilation by Agard *et al.* [111], however, shows good agreement between models and P-T trajectories of recovered rocks.

Despite these challenges, there are subsets of the global array of exhumed subduction complexes that overlap in PT conditions, and that preserve both transient and long-term deformation features that may correlate to the SST source region and process. Studying these exposures can provide key insights into (a) the rock types that occupy this depth range on the plate interface and potential sources of rheological heterogeneity, (b) steady-state and transient deformation mechanisms and modes, (c) effects of metamorphic reactions, and (d) fluid migration patterns and permeability pathways. Here, we summarize some of the primary insights into the SST source region and mechanisms that have been or can be gleaned from studies of exhumed rocks.

### (a) Materials in the SST source region and their significance

**Figure 6.**
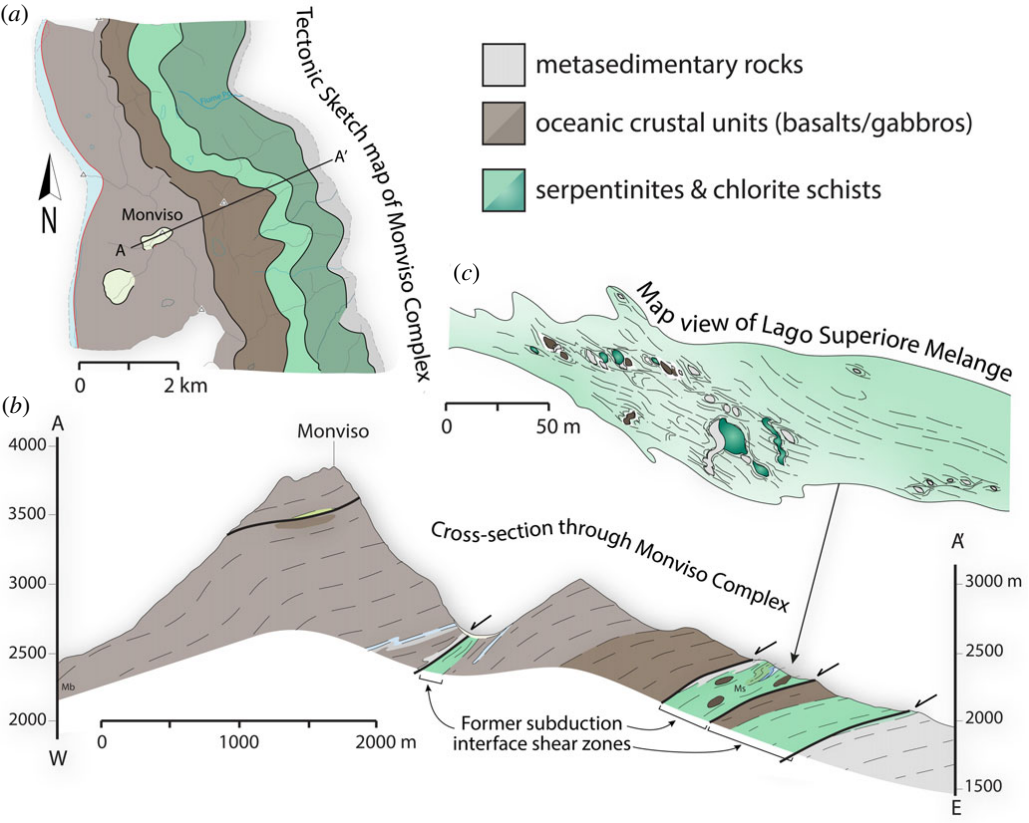
An example of a subducted, underplated and exhumed mafic-ultramafic complex associated with Tethys oceanic subduction preserved in the Western Alps. The complex consists of several slices of basaltic and gabbroic oceanic crust separated by high strain serpentinite and chlorite schist melange shear zones. The melange zones contain blocks of metasediments, eclogite-facies and lower-grade metabasalts and serpentinized peridotites, representing imbricated slivers of oceanic crust and mantle. Tectonic sketch map and cross section modified from [134]. Map view of Lago Superiore melange modified from [135]. (Online version in colour.)

Exhumed subduction complexes tell us which rock types make it to the deep subduction environment without being scraped off in the shallow accretionary prism, and, simultaneously, which rock types become stranded on the deep interface rather than subducting ultimately into the mantle. The process that strands rocks at these depths is referred to as tectonic underplating, which is the progressive transfer of material from the down-going plate to the upper plate and the associated down-stepping of the plate interface (cf. table 1) [112,129–133]. Once individual slices are accreted through the underplating process, they become part of the upper plate forearc crust as the subduction interface migrates downward. Geologic observations of deeply exhumed subduction complexes indicate that sediments, oceanic crustal slices, and mantle slivers are all capable of becoming underplated at and around the SST source depth (figure 6).

Sedimentary protoliths involved in oceanic subduction shear zones typically include cherts, shales, greywackes and pelagic carbonates [136], metamorphosed with progressive subduction to produce schists with variable quartz-mica ratios, meta-cherts (quartzites) and marbles, respectively. Metasedimentary rocks are more abundant in exhumed subduction complexes than expected for their predicted thicknesses on the seafloor, suggesting the deep subduction interface is an important sediment reservoir due to the underplating process [111,137]. The presence of these metasediments has implications for SST, including that (a) they may help explain the correlation between SST and the LVL [30,31,138], (b) they provide an important source of fluid compositions not present in volcanic oceanic crust [139–141], and (c) they are generally rheologically weaker than their mafic counterparts [126,142,143].

**Figure 7.**
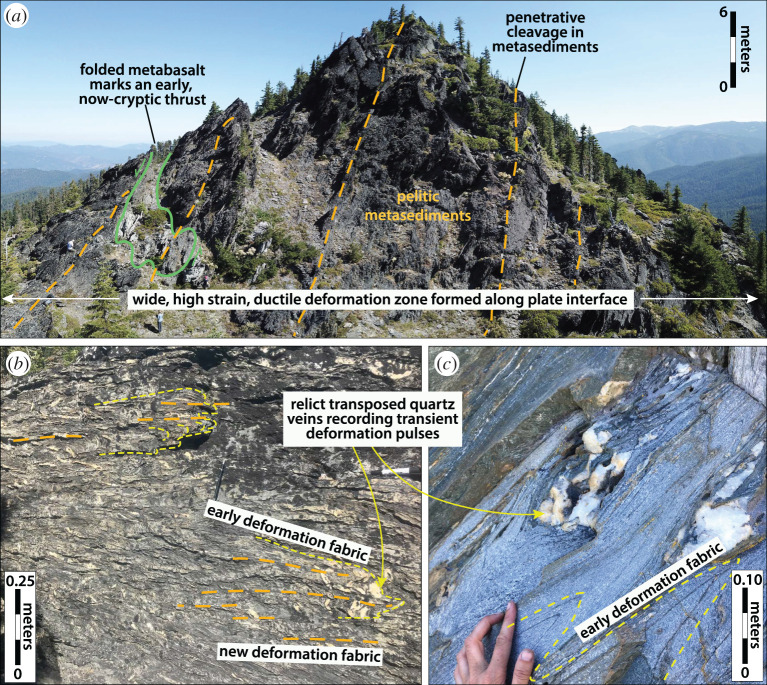
Example of viscous deformation patterns that accumulate over long timescales at SST source depths, from the blueschist-facies (*T* = ∼450^°^C, *P* = 9–11 kbar) Condrey Mountain schist in the Klamath Mountains of northern California/southern Oregon. (*a*) A massif with metasediments tightly folded around a blueschist-facies metabasaltic lens, likely representing an older thrust fault contact, now transposed by distributed viscous deformation. (*b*–*c*) Graphitic mica schists (*b*) and quartz schists (*c*) showing two prograde subduction-related fabrics and several generations of quartz veins variably transposed by the youngest schistosity. While the quartz veins may have formed during transient deformation pulses, the record of this process becomes dismembered due to accumulation of viscous deformation over comaratively long timescales. (Online version in colour.)

**Figure 8.**
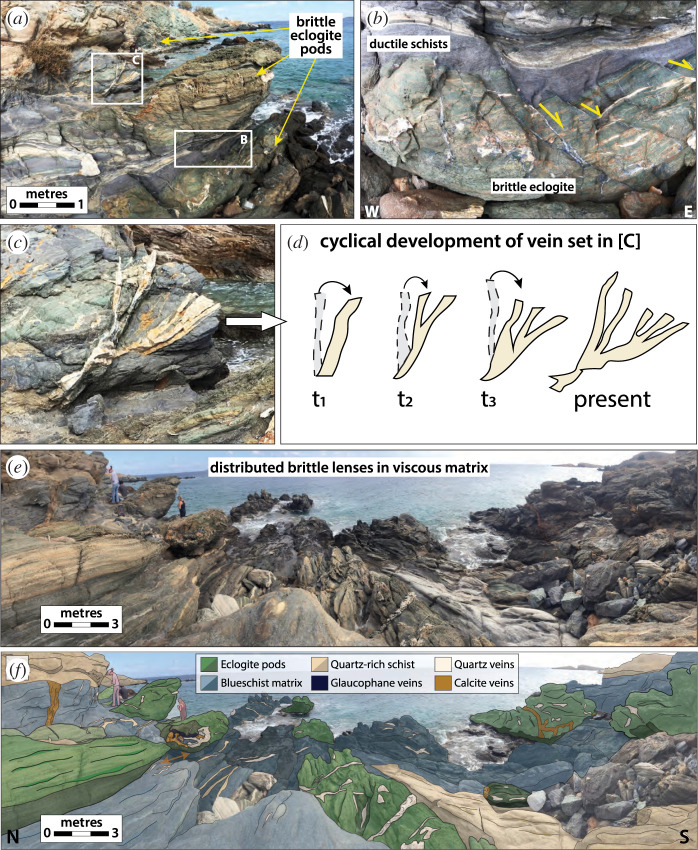
Examples of frictional-viscous deformation in a blueschist-facies shear zone on Syros Island in Greece, modified from [148]. (*a*) Lensoidal mafic eclogite pod (approx. 2.5 m in length) embedded in a blueschist and quartz schist matrix. (*b*) Zoom in on the base of the eclogite pod showing brittle shear veins filled with high pressure minerals (e.g. glaucophane). The brittle shear veins culminate in discrete ductile shear zones in the surrounding blueschist matrix, indicating coeval frictional viscous slip. (*c*) Zoom in on the flanks of the eclogite pod where several generations of quartz veins were emplaced and subsequently sheared in a top-right shear sense, indicating cyclical switches from brittle vein emplacement at high pore fluid pressures to viscous creep and back again (cf. figure 10*a*). (*d*) Sketch illustrating the inferred cyclical development of the quartz vein set in (*c*). The veins show repeated opening and precipitation parallel to Sigma3 (horizontal) followed by progressive rotation in the shear direction. (*e*–*f* ) Panoramic photo of the entire outcrop highlighting the distribution of brittle eclogite pods and their vein structures, within the dominantly viscously deformed matrix. (Online version in colour.)

Oceanic crustal slices are also commonly preserved in deep subduction complexes, with protoliths that include both highly altered and pristine seafloor basalts, as well as intrusive oceanic crustal sequences such as sheeted dykes and gabbros [55,144]. These suggest that various depths of oceanic lithosphere become entrained or sliced off during progressive subduction [129,145,146]. Despite these protoliths all being similar in bulk composition (i.e. mid-ocean ridge basalt), their rheological properties on the subduction interface may change drastically as a function of initial sea-floor alteration, and metamorphic grade. Originally fine-grained, altered basalts, for example, are often tightly folded with pelagic metasedimentary cover sequences on the deep interface (e.g. figure 7), whereas originally coarser-grained gabbroic or unaltered basaltic lenses are more typically incorporated as boudinaged blocks and/or underplated as intact slabs, especially when they have been eclogitized (figure 8) [147–150]. The intact oceanic crustal fragments may be important for SST because they may correspond to multi-kilometre-scale lineaments or smeared `asperities’ on the plate interface that could guide migrating slow slip fronts or tremor bursts in an along-strike and/or downdip direction (e.g. [67,151,152]), depending on interface kinematics and the degree of finite strain accumulated in the surrounding rocks. These mafic fragments may also act as permeability barriers once accreted to the upper plate (cf. §3d and figure 13).

Mantle peridotite slivers (typically serpentinized) are preserved in relatively small volumes in deep subduction complexes and have three potential sources: the down-going slab, supra-subduction zone ophiolites derived from the shallow forearc, and deeper mantle wedge material derived from beneath the upper plate Moho. Derivation from the down-going slab may relate to fracture zones or to abyssal peridotites already exposed on the seafloor, suggesting slow mid-ocean-ridge spreading environments and oceanic core complexes [153,154]; or it may imply especially deep slicing into the down-going slab during underplating [146,155]. Derivation from supra-subduction zone forearc ophiolites or deeper upper plate mantle wedge requires a subduction erosion environment, characterized by entrainment of upper plate material into the subduction shear zone, and usually indicates low sediment supply and/or variations in slab topography [156–159]. The observation that mantle slivers may be incorporated into the subduction shear zone near the trench is important to understanding SST because it implies that ultramafic rocks can be involved as a dehydrating source rock, a weak rheological heterogeneity, and/or a fluid channel or barrier at even shallower depths along the plate interface than the mantle wedge corner.

### (b) Deformation styles and mechanisms on the deep interface

#### (i) Long term deformation patterns

The subduction, underplating and exhumation histories, and the temperatures around and above the brittle–ductile transition within deep subduction complexes, mean that the majority show distributed, polyphase ductile deformation to very high strains. It is common for even the simplest exhumed rocks to preserve three deformational fabrics, including (1) an early prograde fabric cryptically preserved in the cores of later folds and/or as inclusion trails in metamorphic porphyroblasts (e.g. garnet), (2) a penetrative fabric formed at near-peak metamorphic conditions, and (3) a variably developed overprinting fabric produced during later exhumation (e.g. [160–165]). The transition from one fabric to another is often accompanied by changes in kinematics, including tectonic transport direction and/or strain geometry (e.g. [160,166]). Rocks deformed in this distributed ductile manner on the subduction interface can form coherent tracts, continuous across multiple lithological boundaries and previous tectonic contacts (e.g. thrust faults), suggesting that over long timescales the plate interface can be several kilometres in width (figure 7), consistent with multichannel seismic evidence for a thick shear zone in the SST source region [16,167,168]. Even where these coherent terranes have escaped exhumational overprinting, they represent deformation integrated over a minimum of approximately 0.5 Myr (based on maximum subduction rates and slab dips). At these timescales, bulk deformation is accommodated by viscous flow in the weaker subducted units, involving both pressure solution (most common in metasedimentary and fine-grained mafic rocks) and dislocation creep (in some mafic rocks or quartz-rich metasedimentary rocks and veins) [169–173]. Where shear stress magnitudes have been estimated from recrystallized grain size piezometers in quartzites and/or from experimental flow laws, they are typically under 10 MPa [170,174–178]. These long-term deformation patterns are relevant to understanding the SST source region firstly because they reflect deformation processes that dominate over many earthquake cycles, and secondly because of the frozen-in seismic velocity/anisotropy signals they store along the plate interface, which can remain long after the active subduction interface has migrated structurally downward.

#### (ii) Transient deformation patterns

Given the frequency at which SST events recur (days to months, cf. table 3), and that in some localities SST accommodates between 60% and 100% of the total plate boundary slip budget (e.g. [58]), it can be inferred that a significant fraction of the deformation accumulated over million-year-timescales is produced in the short term by SST processes. In making comparisons from the rock record to the SST source region, we are therefore interested in detecting transient deformation signals embedded within the integrated subduction history described above. We distinguish transient features in the rock record as spatial changes in deformation mode, from distributed ductile flow, to synkinematic fracturing, frictional sliding or accelerated viscous creep, which implies a local switch to strain rates elevated above a background steady state [189–195].

**Table 3.** Summary list of relevant source properties of slow slip and tremor (SST) events.

parameter	constraint or observable	typical values and notes	references
**ETS events**			
SSE rupture dimensions	tremor locations and modelling of geodetic data	from few km (tremor only) up to 10 s km wide by 100 s km long	[49,59,65,179]
slip per event	geodetic inversion and tremor calibration	mm to few cm	[49,59,179]
Mw	geodetic inversion (events <∼Mw 5.5 are difficult to detect)	Mw 5.3?–6.7	[49,59]
stress drop	geodetic inversion	1–100 kPa	[49,179]
duration		days to weeks	[64,100,179,180]
recurrence interval		months to years	[41] and references therein
propagation velocity	tremor/LFE space–time distribution	along-strike: 5–15 km d^−1^	[64,67,68,77,181,182]
		along-dip: 30–200 km h^−1^	
		back-propagating: 5–20 km h^−1^	
slip rate	geodesy/tremor	1–2 mm d^−1^	[17,49,69]
rise time (duration of slip at a point on the fault)	geodesy/tremor	5–30 days	[179]
**long-term SSEs**			
SSE rupture dimensions	modelling geodetic data	10 s km wide by 100 s km long	[62,101,183,184]
slip per event	geodetic inversion	up to 10 s of cm	[62,101,183,184]
Mw	geodetic inversion	M6 to M ≪ 7	[62,183–185]
stress drop	geodetic inversion	5–100 kPa	[101]
duration		<100 days, may endure for years	[180,183,184,186]
recurrence interval		2–10 years	[61,101]
propagation velocity	modelling geodetic data	along-strike: 5–15 km d^−1^	
		along-dip: 30–200 km h^−1^	
		back-propagating: 5–20 km h^−1^	[64,67,68,77,181,182]
slip rate	geodesy	1–2 mm d^−1^	[17,49,69]
rise time	geodesy and/or tremor	rise time could be as long as duration, unless individual SSEs represent a sequence of many smaller events (e.g. [63,187])	[62,63,183–185]
**LFEs**			
rupture dimensions	seismic waveforms	0.1–1 km	[6,88]
slip per event	seismic waveforms, number of LFEs per ETS	0.05–0.12 mm	[6,88,92]
		(if multiple slip patches contribute to LFE-family failures, slip could reach few mm) [6,188]	
Mw		1–2.5	[88,92]
stress drop	seismic waveforms	1–10 kPa for 1 km patch size	
		(if dimensions are 0.1 km, stress drops can reach 1 MPa; [181], p. 3364)	[6,88,92]
duration		0.2–0.7 s	[88,92]
recurrence interval (in ‘LFE family’)		seconds to days	[74,91]
rupture velocity		∼0.7 km s^−1^	[92]
slip rate		∼0.25 mm s^−1^	[92]

There are two key challenges in interpreting transient features in the rock record, however. Firstly, *how* elevated the strain rates were is very difficult to quantify from the rock record [196], although some estimates can be gleaned from correlating deformation mechanisms in the rocks to experimental flow laws. The ‘smoking gun’ for recognizing seismic strain rates is pseudotachylite (but see [197] for a summary of other potential indicators of seismically generated frictional heating). Pseudotachylite is described for only one of the 30–50-km-deep exhumed subduction interface localities shown in figure 1 (Corsica [198]), whereas they are described for a handful of subduction complexes exhumed from the shallow subduction interface (e.g. [199–202]). This may imply that fast seismic slip along the deep interface is rare, consistent with seismic observations of modern subduction zones, and with the elevated temperature conditions expected for the deep megathrust. However, the generation of frictional heat is sensitive to both the velocity of slip and the shear stresses, so their absence does not rule out the possibility of seismic slip on the deep interface where shear stresses may generally be very low. The second challenge is that transient deformation features are bound to be only minimally preserved because in many places they are erased by the longer-term aseismic creep process (cf. figure 7).

One of the most widely documented potential markers of transient deformation in subduction complexes are ‘melange belts’, which we loosely define here as localized high strain shear zones in which blocks of higher viscosity material are embedded in a less viscous matrix [203–206]. These most commonly develop in rheologically weak geological units such as phyllosilicate-rich metasediments and serpentinites, and contain mafic or ultramafic slivers or blocks such as coarse-grained amphibolites, eclogites and/or relict peridotites. Experimentally derived flow laws for this range of subduction zone materials predict viscosity contrasts of up to 4 orders of magnitude in the temperature range of SST (see compilation in fig. 2 in [143]). Melange belts are potential culprits for transient deformation firstly because they are characteristically narrower than coherent terranes, implying that they record elevated strain rates, and secondly because the deformation mechanisms they record are nearly always combined frictional-viscous creep at a variety of length scales, e.g. [190,207,208]. That is, due to their high viscosities, the clasts within these melange belts cannot yield viscously and instead accommodate fracture and frictional sliding through veining and faulting that is synkinematic with viscous strain in the surrounding matrix (figures 8 and 12). The brittle deformation in the clasts can be triggered by stress concentrations generated at clast margins and/or by transient increases in pore fluid pressures (e.g. [209–212]). This combined frictional-viscous mechanism of deformation commonly produces S-C-type fabrics similar to those observed in mylonite zones near the brittle–ductile transition in continental shear zones (e.g. [213]).

**Figure 9.**
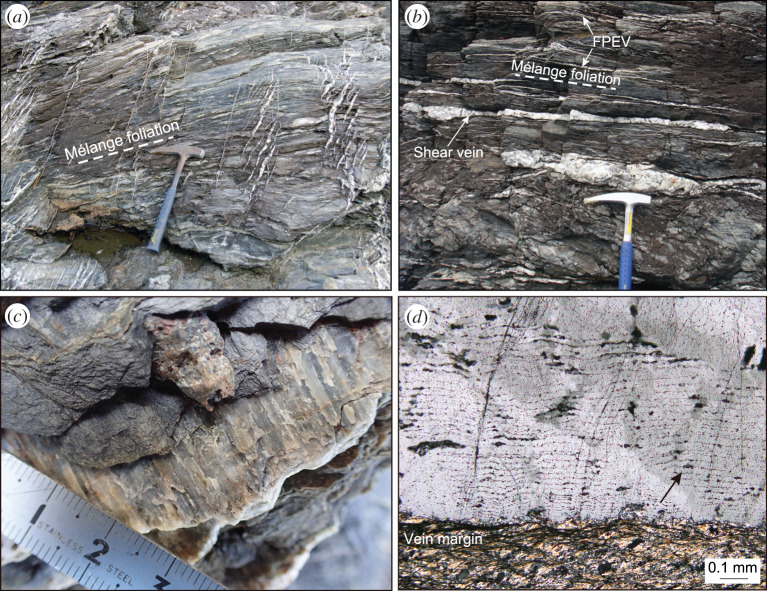
Examples of transient deformation features preserved in the Makimine melange in the Shimanto accretionary complex in Japan, modified from [220]. (*a*) Mutual cross-cutting relationships between melange pressure solution cleavage and extensional shear fractures cf. figure 10*b*. (*b*) Coexistence of foliation-parallel and foliation-perpendicular veins suggesting transient switches in the orientation of sigma-1 with respect to the shear plane. (*c*) Quartz slickenfibres developed on foliation parallel veins highlighting a shear component. (*d*) Photomicrograph of crack-seal texture in foliaton-parallel dilational shear veins, indicating many stages of fracture opening and precipitation. (Online version in colour.)

**Figure 10.**
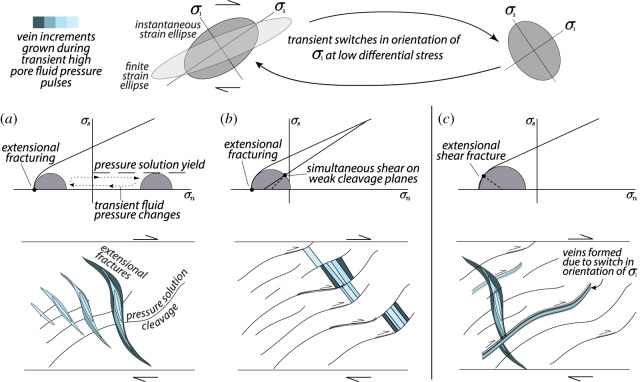
Examples of changes in mechanical state (brittle to ductile) during transient deformation under high pore fluid pressures, as based on vein and fabric relationships in melange belts. (*a*) A scenario in which viscous pressure solution creep dominates long term deformation and controls the orientation of finite strain. Extensional fractures (commonly including a shear component) form during transient high pore fluid pressure pulses, and are subsequently viscously deformed and rotated during inter-event periods (see [148] and cf. figure 8). (*b*) A scenario in which weak cleavage planes formed by viscous creep may evolve into shear fractures oriented at high angles to the maximum principal stress (Sigma-1) due to low cohesive strength and simultaneous high fluid pressures. These shear fractures can be activated at the same time as (and rate-limited by) precipitation into extensional veins (see [190]). (*c*) A scenario in which there are mutual cross-cutting relationships between dilational fractures oriented at both high and low angles to the melange fabric, suggesting transient switches in the orientation of Sigma-1, also an indicator of near-lithostatic pore fluid pressures (see [220] and cf. figure 9). (Online version in colour.)

An additional possible form of transient frictional-viscous creep that does not necessarily require significant lithological variations are localized shear zones that appear to have deformed by some combination of pressure solution, frictional sliding on phyllosilicate laminae or cleavage planes, and dilational micro-cracking [214,215]. Pressure solution involves the dissolution of soluble minerals in the direction of maximum compressive stress and reprecipitation in the extension direction [216–218]. This process segregates insoluble minerals, such as micas, amphiboles and oxides, forming discrete and highly anisotropic cleavage domains, from soluble phases such as quartz precipitated in veins and microlithons at varying angles to the cleavage domains. Several subduction complexes deformed under greenschist and blueschist-facies conditions show evidence for shear slip along weak cleavage planes (e.g. [219,220]) or along kink-like micaceous crenulation bands [175] that appear kinematically linked and coeval with incremental precipitation into dilational fractures (figures 9 and 10).

#### (iii) Transient deformation and fluid pressures

The two types of transient deformation features described above are ubiquitously associated with evidence for cyclical variations in fluid pressures (figure 10). Veins themselves represent tensile fractures, which require the pore fluid pressures to locally exceed the magnitude of the minimum compressive stress [221–223]. The occurrence of purely extensional veins oriented at high angles to the shear fabric in these systems imply approximately lithostatic pore fluid pressures and low differential stresses. It is common for the interior of dilational veins to exhibit ‘crack-seal’ textures that reflect precipitation pulses [219–223,225–228]. In some instances, these show changes in composition of the fluids at approximately constant metamorphic conditions, evidenced by different mineral precipitates from the same metamorphic facies forming in a single vein over time (e.g. [151]). Additionally, in some blueschist and eclogite terranes, garnet zoning patterns appear most consistent with short-timescale growth-dissolution cycles driven by fluid pressure pulses, as opposed to long-term changes in metamorphic conditions (e.g. [228]). Even where crack-seal textures are not present, cross-cutting relationships among vein sets can show a repeating progression of vein opening, infilling, and rotation into the shear plane by ductile creep processes (e.g. figures 8 and 10*a*). There are also examples of mutually cross-cutting relationships between veins opened both perpendicular and parallel to the shear zone fabric, suggesting transient switches in the orientation of the maximum compressive stress, also indicative of very high pore fluid pressures (figures 9 and 10*c*) [220]. These lines of evidence for high pore fluid pressures from the geology are consistent with the geophysical observation of tidal triggering of SST events; additionally, the high fluid pressure is observed over a wide range of metamorphic conditions (greenschist to eclogite facies) and are thus also consistent with the observed high *Vp/Vs* ratios of the seismic LVL discussed in §2a.

#### (iv) Transient deformation length- and time-scales

The transient rock deformation patterns showing a combination of accelerated viscous creep, and cyclical frictional deformation triggered by high fluid pressures qualitatively resemble inferences from geophysical observations of SST (e.g. its correlation with a seismic LVL, high fluid-pressures and low effective stresses inferred from tremor-tide correlations). We can also attempt to make semi-quantitative comparisons of the length- and timescales of transient geologic features relative to SST events.

**Figure 11.**
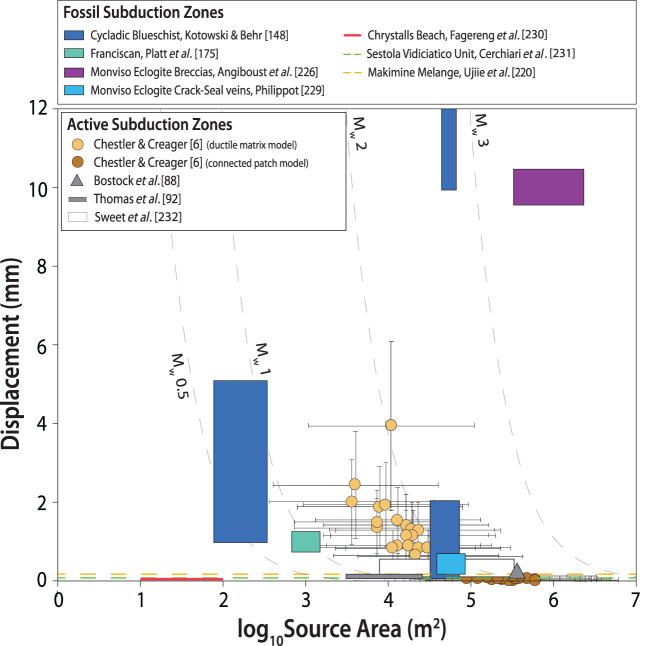
Comparison of source areas and event displacements from active and fossil subduction zones. Displacements from the geological record are based on the widths of crack-seal vein increments and/or measurable shear displacements within shear veins or along faults. LFE source areas and slip are based on the assumption of single-patch failures, except in the models of Chestler and Creager, which assume either 3–10 distributed sources in a ductile matrix or contiguous slippatches make up an LFE-family source area. Source areas are estimated from the mapped area of map-scale heterogeneities (e.g. mafic blocks or melange zones) resolved onto a plane oriented parallel to the dominant subduction foliation (cf. [148]), or from estimates of the length of planar faults or shear zones as described by the authors (in which case equal length and width dimensions were assumed). In some studies, displacements were reported but information on possible source area could not be gleaned—these are plotted as dashed horizontal lines. (Online version in colour.)

First considering length scales, geophysical data suggest the dimensions of individual LFE families is between 100 m and 1 km, and slip-per-event ranges from 0.05 to approximately 3 mm. In geologic exposures, shear and dilational displacements, where recorded by offset features, vein widths or crack-seal textures, are very similar in magnitude to LFE slip (figure 11), suggesting they could relate to the LFE source. However, the slip area of individual veins or shear surfaces in rocks are typically less than 1 m, at least one order of magnitude lower than the minimum size inferred for LFEs from seismology. However, vein sets and shear fractures do commonly cluster in discrete patches, e.g. in shear zones where high viscosity blocks, metamorphic reactions and/or high fluid pressures are concentrated. The length scales of these patches are more compatible with the inferred length scales of LFEs (figure 11). Thus, if we entertain the possibility that displacements within the patches are able to ‘communicate’ over the areas of their geologic exposure, estimated moments are more similar to those inferred from LFEs. Achieving moments characteristic of some of the larger geodetically detected slow slip events would then require this `communication’ process to extend to even larger scales, linking up heterogeneous patches both along strike and up and downdip; or, alternatively, it would require heterogeneous patches of much larger dimension to exist on the deep interface such that what we see in rocks is a minimum length scale due to the exhumation context in which we view them.

In now considering timescales, the geophysical data indicate that recurrence intervals of SST events are on the order of months to years, whereas LFEs belonging to an SST family recur in seconds to days, with hundreds of LFEs within each SST event. As discussed in §2b, LFE recurrence has been interpreted as either repeat failure of a single asperity, or adjoining failures within a relatively tight cluster of asperities. If, as discussed above, we interpret clusters of dilational shear fractures as the geologic record of these failed asperities, healing rate estimates become essential to quantifying likely recurrence times. In the case of block-in-matrix-type melanges, for example vein closure can lead to restrengthening and restoration of cohesion in blocks while simultaneously causing fluid pressure build-ups due to decreased fracture porosity and permeability. And in the case of frictional shear on weak interfaces combined with dilation, the slip process itself will be rate-limited by vein precipitation, e.g. [219]. If very fast healing is assumed, then shear veins opening and closing along a single fault plane with LFE source dimensions may explain LFE recurrence and source. Alternatively, if the LFEs are sourced from heterogeneities within a three-dimensional shear zone, then they may represent distributed dilational shear events that occur rapidly, but in different locations within a thick SST slip zone. In the latter case, vein healing rates could more closely match the recurrence interval of SST events, rather than individual LFEs.

Estimates of healing rates have been examined theoretically, in laboratory experiments, and via natural observations, but proposed timescales for subduction zone settings vary over several orders of magnitude [225,233–235]. Ujiie *et al.* [220] used a kinetic model for quartz precipitation driven by fluid migration [236], and estimated that crack-seal textures in a low temperature (approx. 300^°^C) frictional-viscous shear zone had a minimum healing time of 1.6–4.5 years, generally longer than typical SST recurrence times. This model also predicted a fluid pressure drop of greater than 150 MPa, much larger than the less than 10 MPa estimates of [46]. Recent kinetic models inspired by natural melange shear zones by Fisher *et al*. [237] examined the rates of diffusive redistribution of Si from melange matrix to blocks for two potential driving forces: (1) a transient drop in fluid pressure, and (2) a difference in mean stress between matrix and clasts. Their application of the model to geothermal gradients typical of subduction zones suggests minimum healing times of 10–100 years over the depth range corresponding to SST (approx. 30–50 km), again much longer than SST recurrence intervals, but perhaps more comparable to earthquake recurrence. In contrast to these estimates of annual to decadal timescales, early experiments by Brantley *et al.* [238] and Smith & Evans [233] suggested that crack healing in quartz can be very rapid, completed within hours at temperatures as low as 300^°^C. Additionally, recent work using Li diffusion modelling on transport veins in an eclogite melange block in New Caledonia suggested vein precipitation occurred in one to four months [239]. More estimates like this from experiment and natural observation will ideally help calibrate theoretical models and provide more insight into links between vein precipitation and SST phenomena.

### (c) Geologic constraints on the role of metamorphic reactions

The depth estimates discussed in §2, coupled with the modelled geothermal gradients in modern subduction zones that host SST events suggest that the SST source spans the upper greenschist through blueschist and into lower pressure eclogite and high pressure amphibolite facies for sediments and mafic rocks, and antigorite facies for serpentinized peridotites in the mantle wedge (figure 3) [240]. There are many metamorphic reactions within this range of conditions, several of which involve dehydration and volume reduction [241]. We distinguish two timescales over which these reactions can influence transient deformation patterns that could correlate with seismic phenomena, including SST.

Firstly, reactions themselves can generate instantaneous shear instabilities due to liberation of water [242,243] and/or precipitation of new unstable phases [244,245]. Breakdown of lawsonite and antigorite serpentine, two phases expected to be present in the SST source region, are especially well-studied examples that exhibit instantaneous `dehydration embrittlement’ in laboratory experiments, defined by the development of localized fault planes and/or acoustic emissions [246–250]. Additionally, reaction kinetics experiments on lawsonite and antigorite indicate these reactions are rapid, inducing high fluid discharge rates on the order of 10^−5^ to 10^−8^ s^−1^ (e.g. [251,252]), which is 1–5 orders of magnitude slower than estimated strain rates of viscous relaxation (10^−9^–10^−11^ s^−1^) (e.g. [253]), supporting the idea that dehydration can lead to instantaneous hydrofracture. These reactions have been implicated in both intermediate depth slab seismicity [247,254] and as possible contributors to SST [240,255–258].

To identify instantaneous dehydration embrittlement in the geologic record, we would look for transient deformation features (i.e. highly localized shear zones) in close association with the mineral reaction products. Interestingly, examples of shear instabilities associated directly with lawsonite dehydration reactions in exhumed rocks have thus far not been documented. There are in fact several descriptions of pristine lawsonite pseudomorphs formed on the prograde path with no evidence for closely associated brittle faulting or localized shear strain [259–264]. By contrast, there are a few examples of shear slip generated in close association with antigorite dehydration in the field [245,265]. Some of the best examples come from the Voltri Massif (Erro Tobbio unit) in the Italian Alps where partially hydrated peridotite bodies and serpentinite mylonites exhibit synkinematic shear bands and hydraulic fractures decorated with fine-grained reaction products of antigorite breakdown [266–268]. These may be analogues for tremor signals located in the upper plate of subduction zones near the Moho.

**Figure 12.**
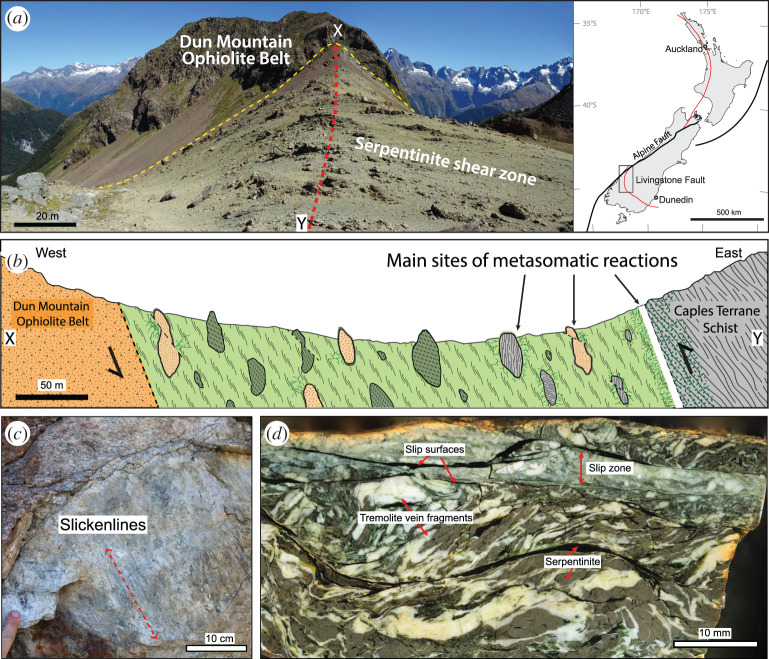
Images modified from Tarling *et al.* [276] illustrating the interplay between chemical reactions and fluid overpressure in a mantle wedge-type setting. The primary metasomatic reactions captured in this shear zone involved the addition of Si and Ca from fluids derived from adjacent metasedimentary schists, producing reaction products such as talc, tremolite, diopside and water. (*a*) Field photo of the 400-m-wide serpentinite shear zone associated with the Livingstone Fault in NewZealand. Inset shows the location of the Livingstone Fault in New Zealand (*b*) Schematic cross section across the shear zone along profile X-Y. The shear zone contains blocks of schist (grey), massive serpentinite (dark green) and rodingite (light orange) embedded in a strongly foliated serpentinite matrix. (*c*) Slickenlines on a frictional slip surface developed within the metasomatic reaction zone. (*d*) Polished slab showing several cataclastic slip surfaces coated with serpentinite cutting across multiple generations of folded and dismembered tremolite veins. (Online version in colour.)

The observation that SSTs do not correlate specifically with a single metamorphic reaction or facies, however, but span several of them (figure 3), suggests that instantaneous shear instabilities cannot uniquely explain these events. Perhaps more compelling is the concept that reactions result in gradual precipitation of new minerals and/or gradual increases in fluid contents and pressures with increasing reaction progress and strain, eventually culminating in transient deformation pulses in the bulk rock. Evidence for this progression is abundant in the rock record and affects a wide range of rock types (and not just in subduction zones, e.g. [269,270]) [271]. The progressive growth, alignment, and concentration of micas in low- to medium-grade subduction interface rocks, for example, has been postulated to strongly influence megathrust seismic behaviours (e.g. [215,272]). Additionally, at the transition from blueschist to eclogite facies conditions in metabasalts, a switch from bulk ductile deformation in the blueschist to brittle deformation in eclogite is commonly observed, with newly formed lenses of eclogite exhibiting fracturing, boudinage, brecciation and/or abundant veining (figure 8) [151,226,273,274]. The switch in deformation mode is interpreted to reflect both a viscous hardening of eclogite relative to its blueschist precursor, and an increase in fluid pressures induced by blueschist mineral dehydration [148,151]. In rocks exhumed from conditions similar to the mantle wedge corner, where peridotites are infiltrated by slab-derived fluids, strain commonly localizes into newly formed, narrow, antigorite shear zones (e.g. [266,275]) and in some places shows evidence for reaction-related fluid overpressures and associated frictional-viscous shear [245,257] (figure 12). The concept of fluid overpressure driven by abundant metamorphic reactions is consistent with the greater propensity for SST events to occur in warm subduction zones, as they release more fluids at comparatively shallower depths than cold subduction zones (e.g. [42,277]).

### (d) The role of fluid migration and permeability

Exhumed rocks from deep subduction environments have proven a rich data source for understanding the role of fluids on the subduction plate interface [278,279]. As discussed in previous sections, there are several prominent sources of fluids anticipated at the SST source depth due to metamorphic reactions in both metasedimentary and mafic/ultramafic rocks, and exhumed rocks unequivocally preserve evidence for abundant fluid activity. Of particular relevance to SST is whether the exhumed rocks preserve information about migration distances and pathways of subduction fluids, and/or spatiotemporal changes in permeability.

Given the recurrence intervals and slip characteristics of SST events (cf. table 3), diffusional fluid flow processes such as grain boundary or volume diffusion are likely too slow to be relevant (e.g. [280]), so we’re most interested in understanding the advection of fluids through vein networks, and along lithological contacts or shear zones, which, as discussed in §3b, are abundant in exhumed subduction complexes. Both structural and geochemical observations shed light on this process. Some exhumed rocks show clear cross-cutting relationships and mineral assemblages among vein networks, such that different generations can be used to estimate the structural permeability of the rock mass under varying metamorphic conditions [194,281–283]. Vein textures themselves also provide some clues, with ‘transport veins’ showing sharp interfaces with the host rock, in contrast to *in situ* dehydration veins, which exhibit diffusional depletion halos at their margins [279,284]. Major and trace-element data and isotopic compositions can also be used to establish whether fluids represented by veins were derived from local dehydration (implying limited transport) or from external sources (implying significant fluid transport) [278,279].

**Figure 13.**
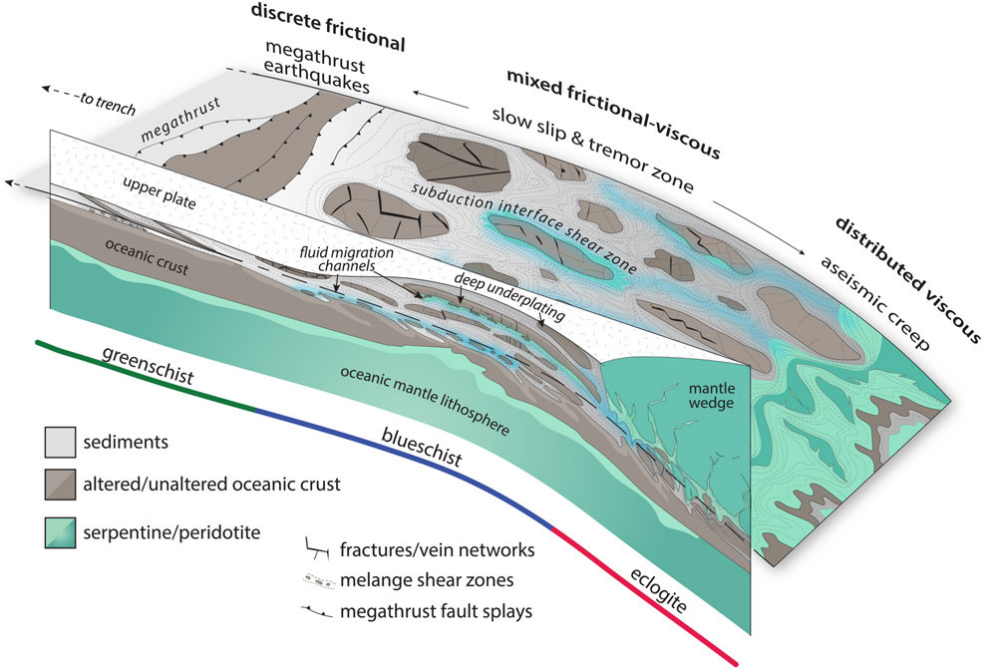
Summary schematic view of the subduction plate interface as inferred from the exhumed geologic record. (Online version in colour.)

An intuitive, first-order observation that emerges from these complementary methods is that open-system fluid–rock interactions and km-scale fluid migration is much more prominent for localized high strain shear zones and melange belts than for coherently underplated mafic terranes that become attached to the upper plate forearc crust above the interface (figure 13) [278,285–287]. In subduction complexes where weakly deformed slices of oceanic lithosphere metamorphosed at blueschist and eclogite facies are exhumed, for example, they dominantly show evidence for local fluid circulation and fluid entrapment [288–294], although there are some examples of fluid focusing into higher permeability transport veins [295]. Indicators of large-scale open system behaviour or fluid transport into or out of weakly or undeformed mafic slabs are limited though, and the few documented examples suggest fluid migration length scales only of the order of tens of metres [296,297]. These relationships thus far imply that underplated and metamorphosed oceanic crustal slices occupying the deep forearc generally act as barriers to fluid flow on the deep interface; they may therefore form transient or long-lived fluid pressure seals.

In many subduction complexes, however, previously underplated and eclogitized mafic fragments become incorporated within the subduction shear zone during exhumation from eclogite through greenschist facies conditions. Once incorporated, these oceanic fragments tend to gradually lose their impermeable quality as they begin to fracture, boudinage and disperse into the shear zone matrix [285,287,298,299]. They may still retain lower permeabilities than the surrounding matrix rocks, however, and can therefore produce large spatial gradients in fluid flux, with the largest fluxes produced in the matrix adjacent to blocks with long-axes oriented parallel to the foliation [285]. Evidence for metasomatism by an external fluid source in these melange or block-in-matrix shear zones abounds. Some key indicators include: (a) substantial addition of Si relative to the melange host rocks, usually in the form of multiple generations of veins of varying orientations to the shear zone foliation [283]; (b) the development of reaction rinds (a.k.a. ‘blackwall alteration zones’) around melange blocks [286,300,301]; and (c) isotopic homogenization of the melange matrix [302].

The phyllosilicate-rich matrix materials that compose melange belts and subduction shear zones are also notoriously anisotropic such that these shear zones dominantly host fluid flow in the plane of the foliation, approximately parallel to the plate interface, therefore promoting fluid migration to lower pressure regions updip [38,303]. Experiments on antigorite serpentinite, for example, show at least one order of magnitude higher intrinsic permeability parallel to foliation than normal to it [38]. Updip fluid flow can also be traced in the rock record by linking fluid geochemistry preserved in veins to deeper metamorphic reactions from which the fluids were liberated. Angiboust *et al*. [287], for example, used major and trace-element data from metasomatized eclogites from the Lago Superiore Unit in the western Alps to argue for approximately 20–30 km of updip flow of fluids sourced from antigorite breakdown reactions downdip. Nishiyama *et al.* [304] recently documented high salinity, high ^3^He/^4^He fluids in strongly sheared greenschist-facies metasediments, also interpreted to represent mantle derived fluids transported updip from the mantle wedge.

Combined, the observations above suggest that underplated tracts of mafic oceanic fragments attached to the forearc crust, and associated meta sediment- and/or serpentinite-rich melange belts that occupy interface shear zones, are likely to strongly suppress vertical fluid flow, such that elevated pore fluid pressures are expected to be at least transiently sustained along the interface.

### (e) A geological model of the SST source region

Figure 13 provides a synthesized view of the subduction plate interface as inferred from the exhumed geologic record and integrated with the geophysical observations discussed in §2. The interface is depicted as grading downward from a discrete megathrust fault and associated brittle fault splays to a wider, distributed, frictional-viscous shear zone and eventually to a zone of fully viscous shear. Through time, the active portion of the subduction interface migrates downward such that earlier subducted material is left stranded above the actively deforming zone—these underplated terranes may control the seismic velocity signatures and/or permeability structure of the broader subduction interface. The deep subduction shear zone can entrain fragments of the downgoing slab, including sediments, altered and unaltered oceanic crustal fragments, and variably serpentinized peridotite. Metasedimentary rocks tend to deform as broadly distributed viscous tracts over long timescales, but they record evidence for transient deformation under high Pf. Mafic rocks commonly form coherent pods or tabular lenses that accommodate transient brittle deformation triggered by fluid pressure cyclicity and stress concentrations at their margins. Serpentinized mantle material, whether entrained from the down-going slab or the upper plate mantle wedge, form high-strain shear zones that show evidence for anisotropic, updip fluid-flow and high fluid pressures accentuated by abundant chemical reactions. In this model of the interface, tremor and LFEs could concentrate in low vertical-permeability, frictional-viscous melange belts where fluids are trapped and rocks types are mixed. LFE migration and streaking along-dip and along-strike during ETS events could be controlled by the distribution of deformed and underplated mafic lineaments. Long-term SSEs without tremor could represent regions in which the frictional heterogeneities in melange belts are too small or widely distributed to permit detection of LFE sources.

## 4. Summary

The preceding overview of the geophysics and geology of the deep subduction interface illustrates the considerable progress being made toward understanding the structure, materials and environment of deep-seated episodic tremor and slow slip (cf.figures 2 and 13). The geophysical record illuminates the close spatial and temporal relationship between low frequency earthquakes and slow slip events, the fluid-rich and high-fluid-pressure habitat that these forms of unconventional seismicity occupy, and the length scales, timescales and mechanisms that define SST slip processes. The geological record strongly supports the concept of abundant fluid migration and high fluid pressures over a range of depths along the plate interface, which may correspond well to the observations of tremor-tide correlations that require very close to lithostatic pore pressure, and the very high *Vp*–*Vs* ratios that also indicate high pore fluid pressures observed in modern subduction zones. Additionally, the most prominent form of transient deformation preserved in rocks from this environment involves an interplay between frictional and viscous mechanisms modulated by cyclical fluid pressure variations and defined by combined shear and vein precipitation. Although each individual transient deformation feature preserved in the rock record is small, the displacements they record, and the total outcrop areas where they are clustered, scale reasonably well with inferred seismic moments of low frequency earthquakes. Key uncertainties and questions remain, however, including the following.

(1) Do LFEs represent repeated (over timescales as short as minutes) rupture of small asperities on a single fault plane or are they distributed over a finite-width shear zone? In the former case, which structures would constitute these discrete fault planes in the rock record? In the latter case, what mechanisms (e.g. fluid–pressure diffusion, viscoelastic stress transfer) allow transient deformation features to communicate within a more distributed shear zone to form a coherent LFE ‘patch’, and to reliably participate in frequently recurring SST events that propagate over 10s of km distances? Addressing these questions would require further improved LFE locations from geophysics, and better constraints on interface shear zone matrix rheology, fluid migration rates and vein precipitation timescales from geology and experiment.
(2) What geological processes distinguish the diverse timescales and recurrence intervals of LFEs and SSEs? What is the relative importance, to fluid pressure buildup and associated valve-like fault behaviour, of processes that *generate* additional fluids (e.g. metamorphic reactions, updip fluid flow), versus those that *trap* fluids in place (e.g. vein closure, shear-induced permeability changes)? Improved estimates of reaction kinetics, rates of fluid flow, and rates of vein mineral precipitation could help us link these various processes to specific timescales within the diverse spectrum of slow slip transients.
(3) Are the characteristics we summarize for the SST depth range unique to the deep interface? Although we focused here on the deep SST zone, tremor and slow slip on the shallow subduction interface of some subduction zones share characteristics with those observed deeper (e.g. [305–307]). However, shallow slow slip is also often associated with abundant microseismicity, indicative of a different thermal environment, contrasting dimensions and physical properties of frictional heterogeneities, and/or variations in local seismic attenuation (e.g. [308]). Nonetheless, the geologic record preserves many of the same features updip of the locked megathrust as it does below it—e.g. wider, more distributed interface shear zones, mixed-lithology melange belts and abundant veining triggered in the shallow case by escape of pore waters and dehydration of clay minerals. Could slow slip and tremor simply be the `motion and sound’ of distributed, rheologically heterogeneous deformation?
(4) What distinguishes short slow slip events that are accompanied by abundant tremor and LFEs (e.g. as observed ETS in Cascadia and Nankai) from long-term, sometimes `tremorless’ SSEs (e.g. as observed in New Zealand)? And what distinguishes portions of ETS slip zones that apparently produce little or no tremor (e.g. [17,182])? Are the different behaviours due to differences in fluid pressures, rock deformation mechanisms, and/or distributions and sizes of mechanical heterogeneities? Are tremor/LFE sources not present in these instances, or are they simply too small to detect?
(5) Can the wide range of geophysical and geological observations inform the development of meaningful new approaches to modelling fault slip in the SST zone? Ultimately, improved constraints on what’s down there; i.e. the rocks, macro- and micro-structures, fluids and reactions, need to be distilled down into meaningful fault-zone model parameters, such that we can better represent the (time-dependent) boundary conditions and geometry of the ETS system. Detailed information about the dynamic evolution of slow slip and tremor failures and their response to changing conditions and external forcing should allow for improved characterization of the spatio-temporally variable deformation, dominant deformation processes and relevant rheological properties.

## Data accessibility

This article has no additional data.
